# Nomogram for prediction of prolonged postoperative ileus after colorectal resection

**DOI:** 10.1186/s12885-022-10377-x

**Published:** 2022-12-06

**Authors:** Zhenmeng Lin, Yangming Li, Jiansheng Wu, Huizhe Zheng, Chunkang Yang

**Affiliations:** 1 Department of Gastrointestinal Surgical Oncology, Clinical Oncology School of Fujian Medical University & Fujian Cancer Hospital, Fuzhou, 350014 Fujian Province China; 2 Department of Gastrointestinal Surgical Oncology, Fujian Provincial Hospital, Fuzhou, 350001 Fujian Province China; 3 Department of Anesthesiology Surgery, Clinical Oncology School of Fujian Medical University & Fujian Cancer Hospital, Fuzhou, 350014 Fujian Province China

**Keywords:** Resection, Prolonged postoperative ileus, Nomogram, Risk factor

## Abstract

**Background:**

Prolonged postoperative ileus (PPOI) is a major complication in patients undergoing colorectal resection. The aim of this study was to analyze the risk factors contributing to PPOI, and to develop an effective nomogram to determine the risks of this population.

**Methods:**

A total of 1,254 patients with colorectal cancer who underwent radical colorectal resection at Fujian Cancer Hospital from March 2016 to August 2021 were enrolled as a training cohort in this study. Univariate analysis and multivariate logistic regressions were performed to determine the correlation between PPOI and clinicopathological characteristics. A nomogram predicting the incidence of PPOI was constructed. The cohort of 153 patients from Fujian Provincial Hospital were enrolled as a validation cohort. Internal and external validations were used to evaluate the prediction ability by area under the receiver operating characteristic curve (AUC) and a calibration plot.

**Results:**

In the training cohort, 128 patients (10.2%) had PPOI after colorectal resection. The independent predictive factors of PPOI were identified, and included gender, age, surgical approach and intraoperative fluid overload. The AUC of nomogram were 0.779 (95% CI: 0.736–0.822) and 0.791 (95%CI: 0.677–0.905) in the training and validation cohort, respectively. The two cohorts of calibration plots showed a good consistency between nomogram prediction and actual observation.

**Conclusions:**

A highly accurate nomogram was developed and validated in this study, which can be used to provide individual prediction of PPOI in patients after colorectal resection, and this predictive power can potentially assist surgeons to make the optimal treatment decisions.

## Introduction

Postoperative ileus (POI) refers to a temporary impairment of gastrointestinal transit due to nonmechanical causes following surgery. As POI occurs in almost all patients following intra-abdominal surgery, especially major abdominal surgery, it may be considered as a normal physiologic response [1, 2]. Usually, it is resolved within 3 days, but may persist or reoccur, in which case it is termed prolonged postoperative ileus (PPOI) [3]. The point at which POI becomes PPOI has not been clearly established. Manifestations of PPOI are characterized as a variable mixture of nausea and vomiting, intolerance of oral diet, abdominal distension and delayed passage of flatus and stool. PPOI is one of the most common complications after colorectal surgery, with an incidence of 3–32% [4, 5]. The variability of reported incidences can be explained by absence of accurate classification criteria and heterogeneous definition of PPOI [6]. PPOI could result in a range of significant consequences, including nutritional deficiencies and the need for parenteral nutrition, increased length of stay, a significant fiscal burden and a negative impact on quality of life which is higher than with other postoperative morbidities [7, 8].

The Enhanced Recovery after Surgery (ERAS) program is an effective and safe protocol, and has been widely implemented in colorectal cancer surgery [9]. Early return of bowel function and prevention of PPOI are important items of clinical practice guidelines for ERAS in Elective Colorectal Surgery [10]. There is currently still a lack of effective treatment options for PPOI, and therefore, it is important to identify high-risk patients of PPOI, and allow early intervention with preventive strategies [11].

Nomogram is a popular and simple tool used to predict the probability of an individual's particular outcome, and has been frequently implemented in clinical practice [12, 13]. The aim of this study was to develop an effective nomogram for prediction of the occurrence of PPOI after colorectal resection.

## Materials and methods

### Patients

One thousand two hundred fifty-four patients hospitalized with colorectal cancer at Fujian Cancer Hospital from March 2016 to August 2021 were enrolled as a training cohort in this study. Patient data were retrospectively accessed from prospectively collected data recorded. The inclusion criteria were as follows: (1) Pathologic diagnosis of adenocarcinoma of the colorectum; (2) Elective radical operation; (3) Age ≥ 18 years. The exclusion criteria were as follows: (1) Preoperative intestinal obstruction; (2) Unassessable on account of dementia or postoperative delirium; (3) Some complications considered to be the cause of PPOI, including postoperative anastomotic leakage, intraabdominal abscess and peritonitis.

**Fig. 1:**
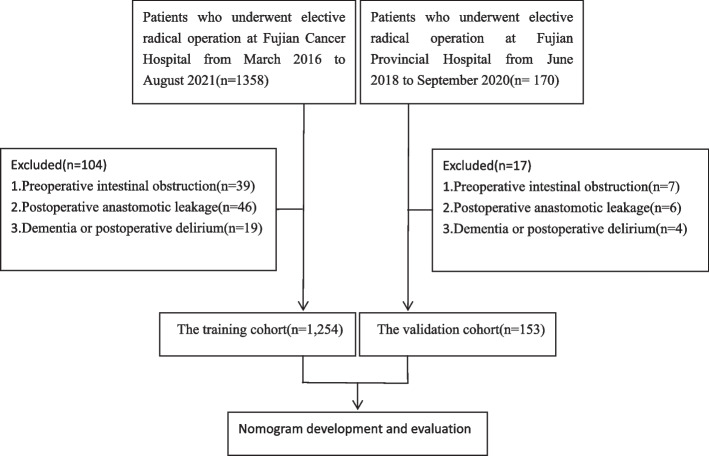
The flowchart of patient selection

One Hundred Fifty-three patients hospitalized at Fujian Provincial Hospital from June 2018 to September 2020 were retrospectively collected from the prospectively maintained institutional database as a validation cohort. The inclusion and exclusion criteria were the same as those for training cohort. The entire flowchart of the selection of patients was depicted in Fig. 1.

### Enhanced recovery program

The ERAS program has been applied in the department of Gastrointestinal Surgery Oncology since November 2014, and has achieved remarkable results. Briefly, our ERAS protocol contains the following: (1) Preoperative period: Preadmission patient education and instruction; Nutritional evaluation and optimization; No routine use of mechanical bowel preparation; carbohydrate loading beverage 12 h and 2 h prior to general anesthesia. (2) Intraoperation period: Minimally invasive surgery is preferred; Intraoperative fluid restriction and avoidance of hypothermia; Nasogastric tubes and drains should be avoided when possible. (3) Postoperative period: Multimodal analgesia; Antiemetic prophylaxis; Early feeding and mobilization [14].

### Definition

PPOI was defined in accordance with the systematic review and global survey by Vather et al. [15]. Specifically, PPOI was diagnosed if patients met ≥ 2 of the following five criteria on POD 4 (postoperative day 4) or later: (1) Nausea or vomiting over the preceding 12 h; (2) Inability to tolerate an oral diet over the prior 24 h; (3) Absence of flatus over the preceding 24 h; (4) Abdominal distention; (5) Radiologic confirmation.

Intraoperative fluid overload was defined as intraoperative fluid replacement ≥ 3 ml/kg/h for laparoscopy and 5 ml/kg/h for open surgery, excluding replacement of blood loss. These cutoffs were independent risk factors for PPOI, and have been identified as critical thresholds for complications in colorectal surgery [16–18].

According to the World Health Organization (WHO) criteria for the adult population, patients were classified as having anaemia based on sex (< 12 g/dl for women, < 13 g/dl for men) [19]. Hypoalbuminaemia was defined as a serum albumin < 3.5 g/dl [20].

### Statistical analysis

Continuous variables with normal distributions are presented as the mean ± SD, and were compared using Student’s t test; Continuous variables with non-normally distributed variables were expressed as medians and interquartile ranges (IQR), and were assessed with Mann–Whitney U tests. Categorical variables were shown as numbers and percentages. The receiver operating characteristic (ROC) curve was used to calculate the best cut-off point of continuous variables. Univariate analysis was performed with a Chi-square test to compare categorical variables. Parameters with significance (*p* < 0.05 in univariate analysis) were selected into multiple logistic regression analysis. R software (version 4.1.1) was used to construct a nomogram based on multiple analysis. The area under the ROC curve (AUC) was calculated to assess the performance of the nomogram. Calibration curves were plotted to compare the predicted probability of the nomogram with the actual probability, while the 45-degree line was used as the perfect model with 100% accuracy. External validation was performed based on the constructed nomogram by validation cohort. The Hosmer–Lemeshow test was used to assess the goodness-of-fit of the model. Statistical analyses were performed through SPSS 26.0 software. A two-tailed *P* value < 0.05 was considered statistically significant.

## Results

### Patient characteristics and outcomes

One hundred twenty-eight patients (10.2%) and 14 patients (9.2%) in the training and validation cohort, respectively had PPOI after colorectal resection. No statistically significant differences in the baseline demographic and the clinicopathological characteristics of the patients were found between the two cohorts (*P* > 0.05), with the exception of hypertension (*P* = 0.027, Table 1).

**Table 1:** Characteristics of patients in the training and validation cohort

	Training cohort (*n* = 1254)	validation cohort (*n* = 153)	*P* value
Gender			0.292
Male	864(68.9%)	99(64.7%)	
Female	390(31.1%)	54(35.3%)	
Age (years)			0.381
≤ 65	625(49.8%)	82(53.6%)	
> 65	629(50.2%)	71(46.4%)	
BMI (kg/m^2^, mean ± SD)	23.4 ± 2.3	23.2 ± 2.2	0.251
Smoking habit			0.298
Yes	312(24.9%)	44(28.8%)	
No	942(75.1%)	109(71.2%)	
Alcohol use			0.633
Yes	532(42.4%)	68(44.4%)	
No	722(57.6%)	85(55.6%)	
Diabetes Mellitus			0.665
Yes	290(23.1%)	33(21.6%)	
No	964(76.9%)	120(78.4%)	
Hypertension			0.020
Yes	354(28.2%)	57(37.3%)	
No	900(71.8%)	96(62.7%)	
Hyperlipidemia			0.419
Yes	402(32.1%)	54(35.3%)	
No	852(67.9%)	99(64.7%)	
Respiratory comorbidity			0.200
Yes	248(19.8%)	37(24.2%)	
No	1006(80.2%)	116(75.8%)	
Cardiac comorbidity			0.535
Yes	244(19.5%)	33(21.6%)	
No	1010(80.5%)	120(78.4%)	
Peripheral vascular disease			0.551
Yes	190(15.2%)	26(17.0%)	
No	1064(84.8%)	127(83.0%)	
Previous abdominal surgery			0.325
Yes	176(14.0%)	26(17.0%)	
No	1078(86.0%)	127(83.0%)	
Preoperative anemia			0.746
Yes	418(33.3%)	53(34.6%)	
No	836(66.7%)	100(65.4%)	
Preoperative hypoalbuminemia			0.342
Yes	200(15.9%)	29(19.0%)	
No	1054(84.1%)	124(81.0%)	
Preoperative WBC count(×10^3^/μL, mean ± SD)	6.8 ± 1.7	7.0 ± 1.6	0.242
Neoadjuvant treatment			0.504
Yes	242(19.3%)	33(21.6%)	
No	1012(80.7%)	120(78.4%)	
ASA-classification			0.395
ASA I	776(61.9%)	86(56.2%)	
ASA II	338(27.0%)	47(30.7%)	
ASA III, IV	140(11.1%)	20(13.1%)	
Surgical approach			0.468
Minimally invasive surgery	1115(88.9%)	139(90.8%)	
Open/conversion	139(11.1%)	14(9.2%)	
Surgical procedure			0.172
Right colectomy	332(26.5%)	34(22.2%)	
Transverse colectomy	78(6.2%)	16(10.5%)	
Left colectomy	330(26.3%)	37(24.2%)	
Rectal resectiona	514(41.0%)	66(43.1%)	
Diverting ileostomy			0.427
Yes	177(14.1%)	18(11.8%)	
No	1077(85.9%)	135(88.2%)	
Operation duration(min)			0.650
≤ 180	574(45.8%)	73(47.7%)	
> 180	680(54.2%)	80(52.3%)	
Intraoperative Blood loss (mL), (median [IQR])	180(90–200)	165(85–200)	0.102
Bowel resection length (cm), (median [IQR])	20(18–21)	20(17,22)	0.113
Anastomosis technique			0.872
Side-to-end	319(25.4%)	38(24.8%)	
End-to-end	935(74.6%)	115(75.2%)	
Anastomosis approach			0.464
Intracorporeal anastomosis	326(26.0%)	44(28.8%)	
extracorporeal anastomosis	928(74.0%)	109(71.2%)	
Intraoperative fluid overload			
Yes	560(44.7%)	64(41.8%)	
No	694(55.3%)	89(58.2%)	
Perioperative transfusion			0.490
Yes	111(8.9%)	11(7.2%)	
No	1143(91.1%)	142(92.8%)	
Differentiation			0.325
Well	65(5.2%)	4(2.6%)	
Moderate	1051(83.8%)	134(87.6%)	
Poor	138(11.0%)	15(9.8%)	
Specimen extraction approaches			0.105
Natural orifice specimen extraction	113(9.0%)	20(13.1%)	
Conventional extraction	1141(91.0%)	133(86.9%)	
TNM stage			0.448
I	213(17.0%)	32(20.9%)	
II	635(50.6%)	76(49.7%)	
III	406(32.4%)	45(29.4%)	

### Univariate and multivariate analysis of PPOI in the training cohort

Clinical characteristics, including gender, age, surgical approach, operation duration, intraoperative fluid overload, were significantly associated with PPOI after univariate analysis (*p* < 0.05, Table 2). Multivariate analysis showed that gender, age, surgical approach, intraoperative fluid overload, were independent predictive factors of PPOI (*p* < 0.05, Table 2).

**Table 2:** Univariable analysis and multivariable logistic regression of clinicopathological variables associated with PPOI

	PPOI, No		*P* value	Multivariate Analysis	
	Absence (*n* = 128)	Presence (*n* = 1126)		OR(95% CI)	*P* value
Gender			0.003	1.933(1.219–3.064)	0.005
Male	103(80.5%)	761(67.6%)			
Female	25(19.5%)	365(32.4%)			
Age(years)			0.001	1.823(1.240–2.679)	0.002
≤ 65	46(35.9%)	579(51.4%)			
> 65	82(64.1%)	547(48.6%)			
BMI (kg/m^2^, mean ± SD)	23.7 ± 2.4	23.3 ± 2.3	0.133		
Smoking habit			0.184		
Yes	38(29.7%)	274(24.3%)			
No	90(70.3%)	852(75.7%)			
Alcohol use			0.282		
Yes	60(46.9%)	472(41.9%)			
No	68(53.1%)	654(58.1%)			
Diabetes Mellitus			0.102		
Yes	37(28.9%)	253(22.5%)			
No	91(71.1%)	873(77.5%)			
Hypertension			0.224		
Yes	42(32.8%)	312(27.7%)			
No	86(67.2%)	814(72.3%)			
Hyperlipidemia			0.428		
Yes	45(35.2%)	357(31.7%)			
No	83(64.8%)	769(68.3%)			
Respiratory comorbidity			0.529		
Yes	28(21.9%)	220(19.5%)			
No	100(78.1%)	906(80.5%)			
Cardiac comorbidity			0.151		
Yes	31(24.2%)	213(18.9%)			
No	97(75.8%)	913(81.1%)			
Peripheral vascular disease			0.348		
Yes	23(18.0%)	167(14.8%)			
No	105(82.0%)	959(85.2%)			
Previous abdominal surgery			0.176		
Yes	23(18.0%)	153(13.6%)			
No	105(82.0%)	973(86.4%)			
Preoperative anemia			0.291		
Yes	48(37.5%)	370(32.9%)			
No	80(62.5%)	756(67.1%)			
Preoperative hypoalbuminemia			0.361		
Yes	24(18.8%)	176(15.6%)			
No	104(81.2%)	950(84.4%)			
Preoperative WBC count( ×10^3^/μL, mean ± SD)	7.0 ± 1.4	6.8 ± 1.7	0.207		
Neoadjuvant treatment			0.210		
Yes	30(23.4%)	212(18.8%)			
No	98(76.6%)	914(81.2%)			
ASA-classification			0.376		
ASA I	72(56.3%)	704(62.5%)			
ASA II	39(30.5%)	299(26.6%)			
ASA III, IV	17(13.3%)	123(10.9%)			
Surgical approach			0.000	2.436(1.519–3.907)	0.000
Minimally invasive surgery	100(78.1%)	1015(90.1%)			
Open/conversion	28(21.9%)	111(9.9%)			
Surgical procedure			0.099		
Right colectomy	38(29.7%)	294(26.1%)			
Transverse colectomy	8(6.3%)	70(6.2%)			
Left colectomy	22(17.2%)	308(27.4%)			
Rectal resectiona	60(46.9%)	454(40.3%)			
Diverting ileostomy			0.432		
Yes	21(16.4%)	156(13.9%)			
No	107(83.6%)	970(86.1%)			
Operation duration(min)			0.047	1.415(0.963–2.078)	0.077
≤ 180	48(37.5%)	526(46.7%)			
> 180	80(62.5%)	600(53.3%)			
Intraoperative Blood loss (mL), (median [IQR])	200(100,250)	180(90,230)	0.063		
Bowel resection length (cm), (median [IQR])	20(18,22)	19(17,21)	0.366		
Anastomosis technique			0.446		
Side-to-end	29(22.7%)	290(25.8%)			
End-to-end	99(77.3%)	836(74.2%)			
Anastomosis approach			0.078		
Intracorporeal anastomosis	25(19.5%)	301(26.7%)			
extracorporeal anastomosis	103(80.5%)	825(73.3%)			
Intraoperative fluid overload			0.001	1.844(1.264–2.690)	0.001
Yes	75	485			
No	53	641			
Perioperative transfusion			0.381		
Yes	14(10.9%)	97(8.6%)			
No	114(89.1%)	1029(91.4%)			
Differentiation			0.820		
Well	7(5.5%)	58(5.2%)			
Moderate	109(85.2%)	942(83.7%)			
Poor	12(9.4%)	126(11.2%)			
Specimen extraction approaches			0.071		
Natural orifice specimen extraction	6(4.7%)	107(9.5%)			
Conventional extraction	122(95.3%)	1019(90.5%)			
TNM stage			0.649		
I	18(14.1%)	195(17.3%)			
II	67(52.3%)	568(50.4%)			
III	43(33.6%)	363(32.2%)			

### Construction of a nomogram for prediction of PPOI

**Fig. 2:**
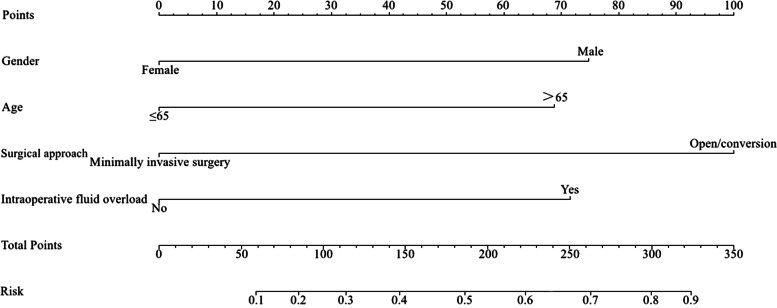
Nomogram to predict the probability of PPOI after colorectal resection. A straight line was drawn vertically from the axis of each variable toward the “Points”scale. The points for each variable were summed together to generate a total point score, which is projected on the bottom line to obtain the individual predictive risk of PPOI

The four variables that were finally determined to be significant in the multivariate logistic regression analysis were used to establish the intuitive nomogram model (Fig. 2). A total score is calculated by summing the scores for each variable, and the final predicted risk of PPOI is the corresponding probability of the total points of individual patients.

### Validation of the nomogram

**Fig. 3:**
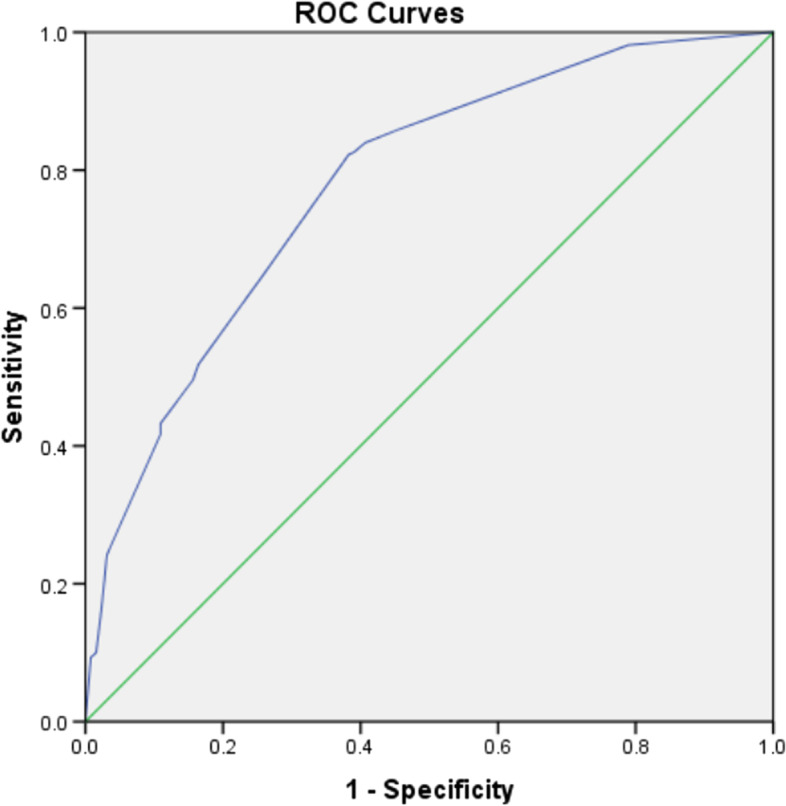
The ROC curve (blue) of nomograms for PPOI in the training cohort

**Fig. 4:**
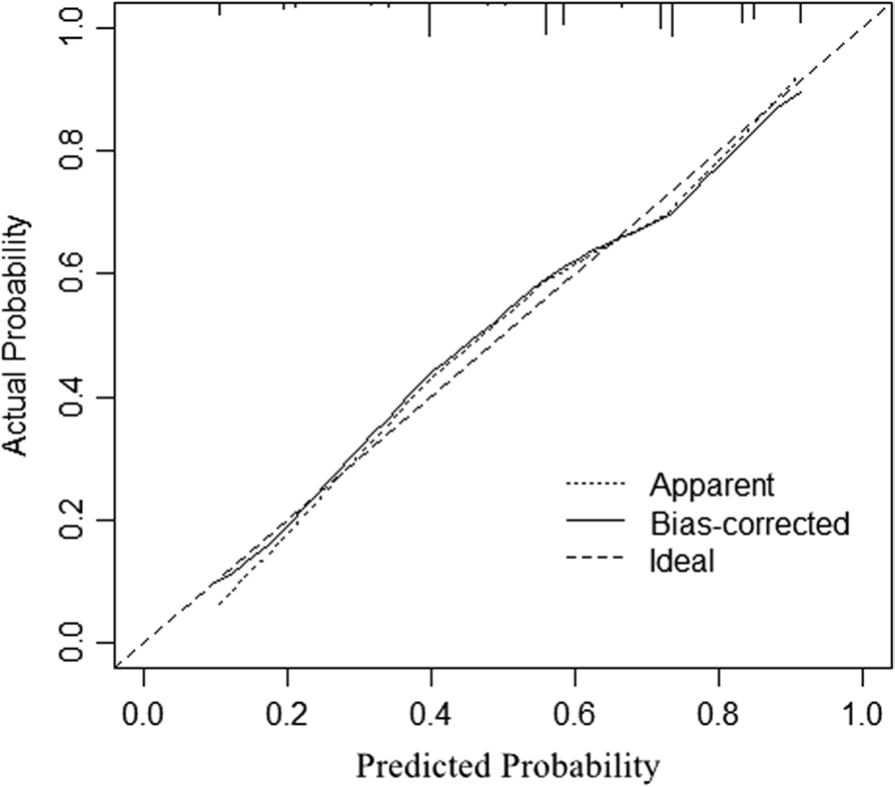
Calibration plots of the nomogram in the training cohort. The apparent line represents actual nomogram performance. The bias-corrected line represents the bootstrap-corrected performance of the nomogram. The diagonal line is an ideal model, indicating 100% predictive power

Internal validation was first performed in the training cohort. The AUC of the training cohort was 0.779 (95% CI: 0.736–0.822) (Fig. 3). A bootstrap resampling procedure was applied and a calibration curve was plotted (Fig. 4). There was good agreement between the predicted and observed probabilities. The Hosmer–Lemeshow test showed an excellent fit (χ^2^ = 5.459, *p* = 0.679).

**Fig. 5:**
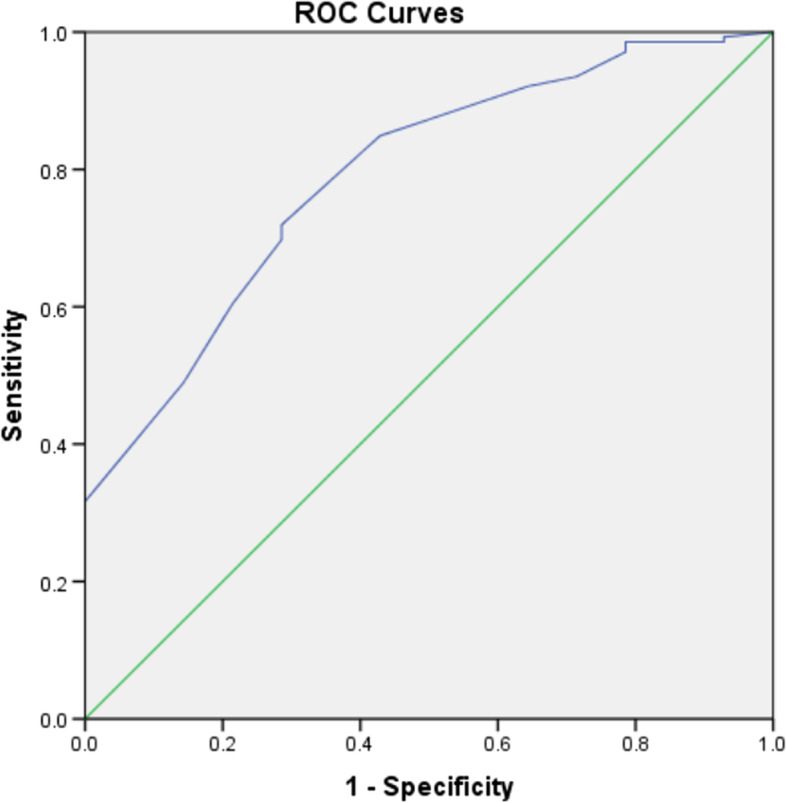
The ROC curve (blue) of nomograms for PPOI in the validation cohort

**Fig. 6:**
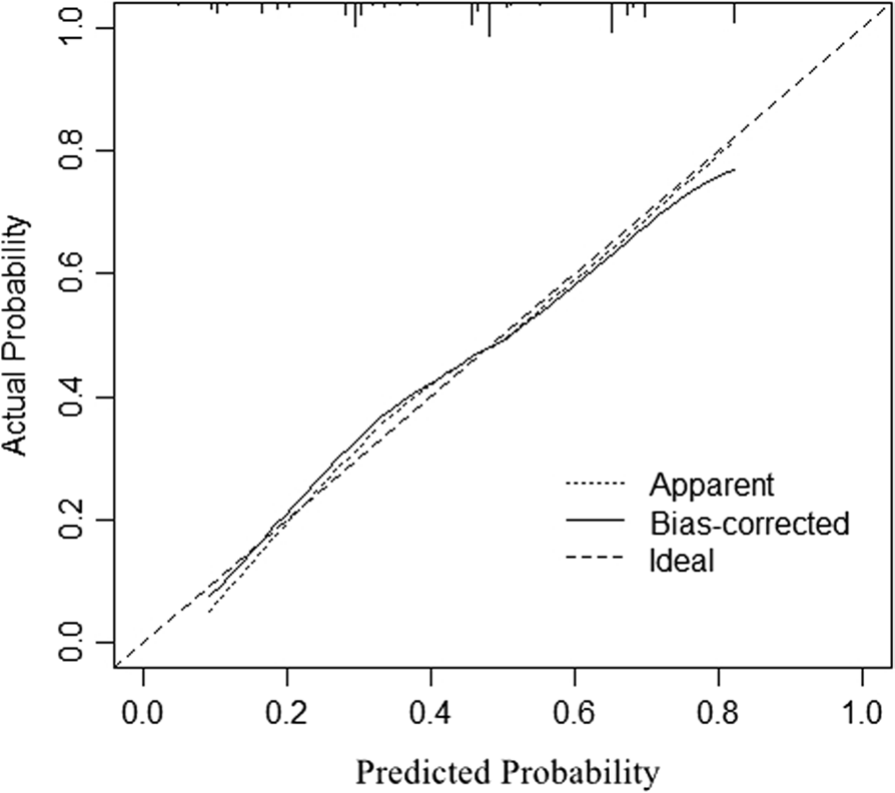
Calibration plots of the nomogram in the validation cohort. The bias-corrected line represents the bootstrap-corrected performance of the nomogram. The diagonal line is an ideal model, indicating 100% predictive power

External validation was further performed in the validation cohort. The AUC was still as high as 0.791 (95%CI: 0.677–0.905) (Fig. 5). The nomogram calibration curve showed acceptable agreement between prediction and actual observation (Fig. 6).

## Discussion

In the present study, there was a 10.2% and 9.2% rate of PPOI in the training and validation cohort, respectively. This incidence was different from previous reports. Wolthuis et al. [3, 4] found the rate of PPOI was 15.9% after colorectal resection and Vather et al. [14] reported that PPOI occurred in 88 of 327 patients (26.9%) undergoing elective colorectal surgery [21]. Liang et al. [22] demonstrated that the overall PPOI rate was 21.5% in 311 patients diagnosed with gastric or colorectal cancer. The difference of incidence was possibly due to ERAS programs have been widely implemented in our study.

Statistically significant differences were found in hypertension between the training and validation cohorts, but hypertension itself was not found to be associated with PPOI. In general, baseline data were essentially balanced in the two cohorts. The AUC of the nomogram was 0.779 in the training. The calibration plots showed a good agreement between nomogram prediction and actual observation, indicating that the model had a good diagnostic performance and an excellent calibration. In addition, the external validation of the nomogram showed a satisfactory outcome, which indicated that our nomogram could be used in various populations and clinical scenarios.

There is no consensus on the independent risk factors. We excluded the secondary PPOI resulting from postoperative anastomotic leakage, intraabdominal abscess and peritonitis [23, 24]. The use of opioid analgesics in the postoperative period was identified as an increased risk for PPOI [25, 26]. However, this information is not available pre/intraoperative and cannot contribute to the prediction model.

An age older than 65 years was identified as an independent risk factor for PPOI. This may be due to the fact that older individuals generally have more medical comorbidity and clinical frailty, and poorer nutritional and functional status compared with their younger counterparts [27]. Our result emphasizes that postoperative surveillance should be especially carefully achieved in such patients who have an increased risk of morbidity and mortality after colorectal cancer surgery [28].

The fact of male sex has also been shown to affect PPOI following colorectal resection. Consistent with the present study, some studies confirmed that the male sex was associated with increased risk of PPOI in elective colorectal surgery [23, 27–29]. This difference is explained by the narrower male pelvis which may make the surgery more difficult and challenging, and potentially secondary to the effects of estrogen and progesterone receptors throughout the gastrointestinal tract and differences in enteric nervous system signaling [17, 30].

Minimally invasive approaches include laparoscopic and robotic surgery. The advantages of robotic surgical systems such as superior instrumentatione and field of vision enable precise dissection in confined spaces such as the pelvis, allowing it to have rapidly gained acceptance in colorectal surgery [31]. The robotic surgical systems for the treatment of colorectal cancer were introduced into this hospital in 2020, but only a minority of patients have been treated with robotic surgery because of its high cost. Previous studies have shown that there are no significant differences between laparoscopic and robotic approaches to PPOI and perioperative mortality [32, 33]. Therefore, we combined laparoscopic and robotic surgery into one group in this study. The surgical approach was the strongest predictor of PPOI in our study. There is high-quality evidence supporting the routine use of a minimally invasive approach to patients with colorectal cancer. Compared with open surgery, minimally invasive surgery has shown better outcomes, including less postoperative pain, shorter time to flatus/bowel motion and oral nutrition, improved cosmesis, less intraoperative blood loss, reduced length of stay, improved cosmesis and similar long-term survival [34, 35].

Previous studies believed that adherence to judicious intra-operative fluid management protocols was protective against development of PPOI [16, 36]. Similarly, this study showed that intraoperative fluid overload was significantly associated with PPOI. This may be because hypervolemic management may result in electrolyte disturbances and splanchnic edema and increased abdominal pressure with decreased mesenteric blood flow, which in turn elicits disruptive tissue oxygenation and ultimately leads to prolongation of the recovery of bowel function [16].

We acknowledge that several limitations still existed in this study. Firstly, the compliance with the ERAS protocol elements cannot be evaluated as this is a retrospective study of prospectively collected data recorded. In addition, the compliance with the protocol has not been unified yet. To date, there are no prospective or clinical trials evaluating the grade of implementation of ERAS. Secondly, the nomogram was developed and validated in only two hospitals rather than multiple centers, thus potentially raising the likelihood of bias. Thirdly, several studies have shown preoperative gut microbiota may be used as biomarkers to predict the development of PPOI [37, 38]. Mucosal samples were not collected in this study, the relationship between them cannot be evaluated.

## Conclusions

PPOI is a common complication after colorectal surgery. Our results have shown that gender, age, surgical approach and intraoperative fluid overload are significantly related to the risk of PPOI. The nomogram with these four factors can accurately predict the probability of PPOI and enable surgeons to guide clinical individualized activities.

## Data Availability

The datasets generated and analyzed in this paper can be made available from the corresponding author on reasonable request.

## Abbreviations

PPOI: Prolonged postoperative ileus
POI: Postoperative ileus
ERAS: Enhanced recovery after surgery
AUC: Area under the receiver operating characteristic curve
IV: Total intravenous
POD: Postoperative day
IQR: Interquartile ranges
ROC: Receiver operating characteristic
